# Gastric hyperplastic polyp with focal cancer

**DOI:** 10.1093/gastro/gou077

**Published:** 2014-10-31

**Authors:** Adam Roman Markowski, Katarzyna Guzinska-Ustymowicz

**Affiliations:** ^1^Department of Internal Medicine and Gastroenterology, Polish Red Cross Memorial Municipal Hospital, Bialystok, Poland; ^2^Endoscopy Unit and Outpatient Department, Hospital of the Ministry of Interior, Bialystok, Poland; ^3^Department of General Pathomorphology, Medical University of Bialystok, Bialystok, Poland

**Keywords:** gastric hyperplastic polyp, gastric cancer, surveillance

## Abstract

This paper reports a rare case of early adenocarcinoma within the gastric hyperplastic polyp, that was completely resected during an endoscopic procedure, and discusses current recommendations in such cases. Endoscopic resection of polyps with focal dysplasia or cancer is commonly indicated, as long as the procedure can be performed safely. After complete excision of a polyp with atypical focal lesion, endoscopic surveillance is suggested. The frequency of surveillance endoscopy should depend on the precise histopathological diagnosis and possibility of confirming the completeness of the endoscopic resection. If the completeness of the procedure is confirmed both macro- and microscopically, gastric resection does not have to be performed. A follow-up esophago-gastroduodenoscopy should be performed at 1 year and then at 3 years.

## Introduction

Gastric polyps are found in the course of approximately 6% of upper gastrointestinal endoscopy procedures and in 1% of autopsy procedures [1]. Until recently, gastric hyperplastic polyps were considered to be insignificant in terms of potential malignant transformation; however, current studies report the presence of adenomas, dysplasia (intraepithelial neoplasia) and even invasive cancer in some gastric hyperplastic polyps [2, 3].

## Case presentation

Prior to a scheduled valve replacement procedure, a 60-year-old female patient with severe aortic valve stenosis was referred by her cardiac surgeon to the Gastroenterology Outpatient Clinic for diagnosis of anaemia, and subsequently admitted to the Department of Gastroenterology of our hospital for the resection of gastric hyperplastic polyps with focal dysplasia.

**Figure 1.**
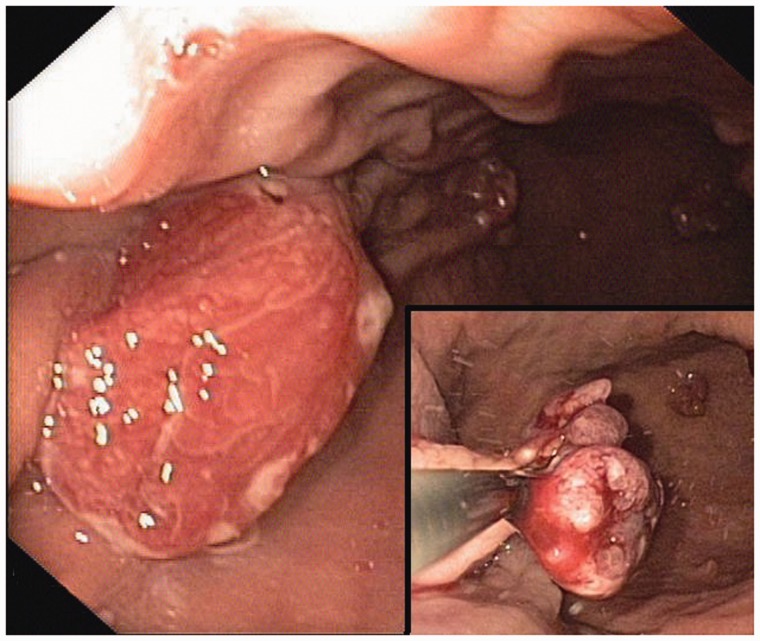
Endoscopic view. Large gastric hyperplastic polyp with focal dysplasia, which turned out to be the focus of early adenocarcinoma. Endoscopic snare polypectomy.

During the ensuing esophago-gastroduodenoscopy (EGD), the largest polyp—sized 25 mm, situated in the gastric corpus and containing focal intraepithelial neoplasia—was resected. Using a submucosal saline injection technique for the reduction of iatrogenic thermal injury—and to increase the probability of a truly curative complete resection—the polyp with focal dysplasia was lifted up and removed *en bloc* with the diathermic loop. This radical endoscopic resection exposed the *muscularis propria* layer (Figure 1). At the end of the procedure, the patient, who belonged to a group at high-risk of cardiovascular complications, developed symptoms of vasovagal syndrome with secondary bradycardia (heart rate = 40/min) and hypotonia (blood pressure = 95/60 mmHg). The procedure was stopped, and the symptoms resolved following fluid infusion and atropine administration.

During a second endoscopic procedure, performed one month later, the four more polyps, ranging in size from 7 mm to 20 mm, were resected from the mid gastric corpus and in a third procedure the last four polyps were excised from the antrum.

The second and third stages of gastric polyp removal were uncomplicated. All polyps were completely resected endoscopically.

**Figure 2.**
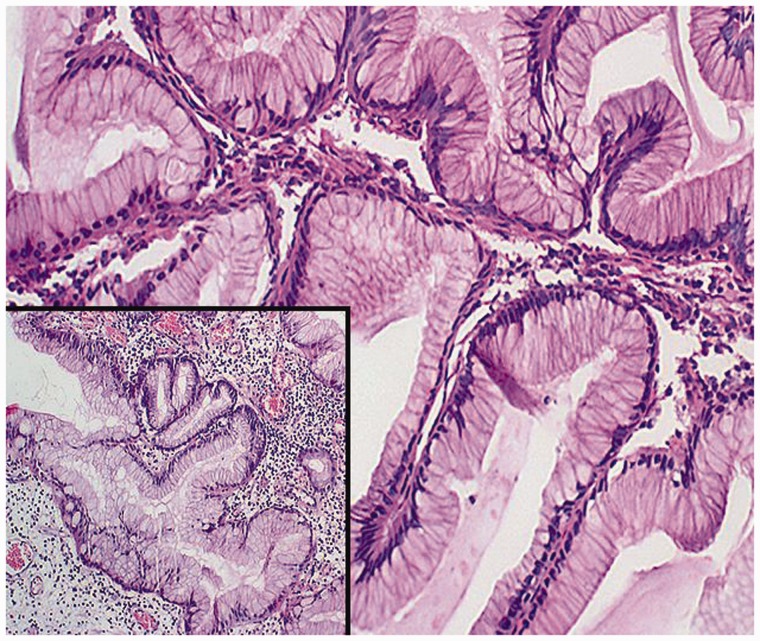
Histopathological findings (hematoxylin-erosin staining, x20/x40). Typical features of gastric hyperplastic polyp with marked elongation of the pits with branching, resulting in a corkscrew appearance.

**Figure 3.**
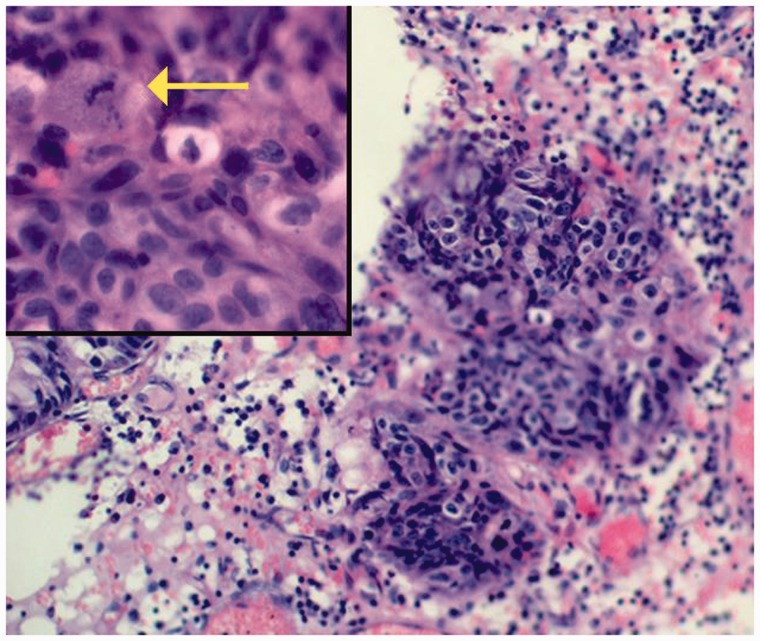
Histopathological findings (hematoxylin-erosin staining, x20/x40). Small focus of adenocarcinoma in the gastric hyperplastic polyp, with a distinct mitotic figure (arrow).

**Figure 4.**
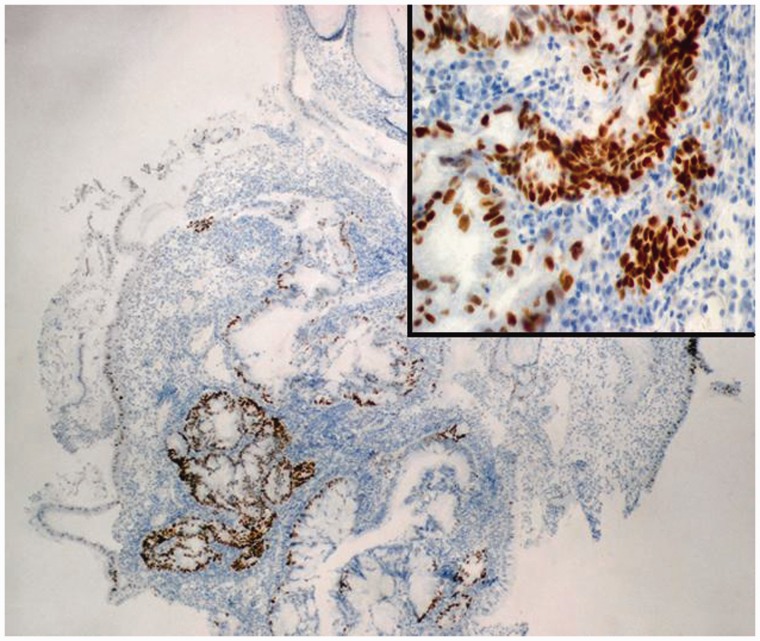
Immunohistochemical findings (x20/x40). The strong positive expression of p53 protein in the focus of adenocarcinoma within a gastric hyperplastic polyp.

Histopathological evaluation of the largest hyperplastic polyp resected from the body of the stomach (Figure 2) revealed well-differentiated focal adenocarcinoma (Figure 3) with significant expression of p53 protein (Figure 4). The lesion did not extend beyond the *muscularis mucosae*, were completely removed endoscopically with an excision margin of greater than 2 mm, and no evidence of lymphovascular invasion was identified. Accordingly, mucosectomy was microscopically assessed as complete. The polyp with focal adenocarcinoma was classified as early stomach cancer, grade 0–Is (superficial, protruded, sessile) according to the Paris Classification of Superficial Gastrointestinal Neoplastic Lesions, and grade T1a by the TNM Classification of Malignant Tumours. Histopathological assessment of biopsy specimens, collected from the mucous membrane of the antrum and the body of the stomach, showed chronic gastritis as well as *helicobacter pylori* (*H. pylori*) infection; eradication therapy was prescribed.

## Discussion

This paper describes a rare case of adenocarcinoma in gastric hyperplastic polyp in a patient with chronic gastritis and *H. pylori* infection. Hyperplastic polyps are usually asymptomatic, typically revealed accidentally during endoscopic examination. In this particular case, diagnosis of microcytic anaemia was an indication for EGD. Its aetiology could involve the fragile, periodically bleeding polyp tissue with tiny surface erosions, as well as chronic gastritis secondary to *H. pylori* infection (the bacteria may compete with the host for iron ions). Heyde's syndrome needs to be considered in the differential diagnosis (gastrointestinal bleeding from colonic angiodysplasia, caused by the acquired deficiency of unusually large von Willebrand factor multimers, secondary to aortic valve stenosis) [4]. Laboratory tests did not show coagulation abnormalities and the performed colonoscopy revealed the presence of only a few small diverticuli within the sigmoid colon. The focal intraepithelial neoplasia (at least low-grade) was found in the analysed tissue specimens of the largest gastric hyperplastic polyp, which was an indication for polypectomy.

The resection of symptomatic polyps, as well as polyps with atypical focal lesions or larger than 1–2 cm, is commonly supported, due to the high potential for malignant transformation [5]. The procedure is more likely to be completed by *en bloc* polyp resection, using the diathermic loop. Typical post-procedural management of patients undergoing endoscopic mucosal resection (EMR) in the stomach includes fasting on the day of procedure and a liquid diet from the first post-operative day onwards. It takes the post-EMR ulceration approximately 6–8 weeks to heal completely. Until then, the patient should be prescribed proton pump inhibitors and potentially also sucralfate. Our patient received omeprazole i.v. before the procedure, in order to improve the conditions for haemostasis in the stomach, later followed by an oral omeprazole treatment regimen for 8 weeks.

Additionally, gastric mucosal specimens (from outside the polyp) should be collected in order to confirm *H. pylori* infection and search for potential atypical and/or pre-cancerous focal lesions. The targeted biopsies of all endoscopically observed lesions and non-targeted biopsies should be performed routinely according to the Sydney protocol. Five separate tissue specimens should be collected: two from the pre-pyloric area (from the lesser and greater curvature, at approximately 3 cm proximally to the pylorus), two from the body of stomach (from the lesser and greater curvature, at 8 cm distally to the cardia) and one from the *incisura angularis* [6, 7].

A single follow-up EGD at 1 year is usually indicated after resection of gastric hyperplastic polyps [8]. There are currently no indications for oncological endoscopic surveillance in cases of gastric hyperplastic polyps without dysplasia. Another follow-up schedule applies to confirmed cases of dysplasia or cancer in a polyp; however, it is commonly held that cancer hardly ever develops in hyperplastic gastric polyps; therefore they are not considered to be a pre-cancerous condition. Yet it is known that the risk of cancer increases with polyp size, having been assessed as significantly greater for hyperplastic polyps exceeding 2 cm, although cancer has been reported in polyps as small as 5–10 mm [8].

It is thought that hyperplastic polyps develop in response to chronic gastritis. According to Orlowska *et al*., the risk of cancer in the gastric mucosa outside the polyp is slightly higher than in the polyp itself [2], probably due to the development of gastric hyperplastic polyps secondary to the known precancerous conditions, such as chronic gastritis associated with *H. pylori* infection or chronic atrophic gastritis. Once *H. pylori* infection—being one of potential underlying causes of gastric hyperplastic polyps—is eradicated, a significant percentage (<70%) of gastric hyperplastic polyps regress in 1–12 months [9].

According to Cao *et al*., the incidence of gastric hyperplastic polyps fell between 2000 and 2010 (48.5 *vs.* 20.8%, respectively), probably due to the lower incidence of *H. pylori* infections (54.4 *vs.* 37.7%, respectively) [10]. In a Polish study by Orlowska *et al*., the incidences of dysplasia and cancer in 483 gastric hyperplastic polyps were 3.3% and 2.1%, respectively [2]. In histopathological evaluation of 497 hyperplastic gastric polyp specimens collected from Japanese patients, the incidence of dysplasia in polyps was 10%, and the incidence of cancer was 2.2% [3]. In the last study [3], all cases of cancer in a gastric hyperplastic polyp showed the expression of p53 protein and high mitotic index of Ki-67. The focal intraepithelial neoplasia was always situated close to focal adenocarcinoma, which appears to confirm the theory of malignant conversion of hyperplastic polyps, i.e. hyperplasia –dysplasia–adenocarcinoma. The studies comparing the results of histopathological evaluation of biopsy specimens and polyps resected *en bloc*, show approximately 90% consistency. Large polyps are resected, not only for the purposes of precise histopathology-based grading, but also for prevention of neoplastic transformation. Precise determination of the grade of dysplasia is of the utmost importance as a predictive factor for malignant conversion and the risk of metachronic stomach cancer. The observed annual dysplasia (also intraepithelial neoplasia) progression rate is highly variable, ranging between 0–73%. Clinical studies show that the preliminary diagnosis of low-grade focal dysplasia in histopathological specimens—collected using biopsy forceps from macroscopically abnormal areas during endoscopy—may be verified, and the grade of atypia increased to high-grade dysplasia or even cancer, if the lesion is totally resected during EMR. According to some authors, as gastric hyperplastic polyps may contain adenoma, intraepithelial neoplasia or even adenocarcinoma, and the biopsy specimen analysis results may be false-negative, all gastric polyps exceeding 20 mm (or, according to some opinions, even 5 mm) should always be resected [7]. Lymph node involvement and haematogenous metastases of early gastric cancer limited to the mucosa are, respectively, rare and extremely rare at the time of diagnosis,. Only lymph node metastases are observed in clinical practice [11].

Our patient had no family history of gastrointestinal or colorectal malignancies or polyposis syndromes. Gastric hyperplastic polyposis is a rare, inherited autosomal dominant syndrome, characterized by the presence of ≥50 gastric hyperplastic polyps and often associated with colorectal neoplasm [12, 13]. Total gastrectomy with lymphadenectomy is justified in patients with diffuse involvement of the stomach by polyps, which can make detection of a synchronous focus of cancer difficult [14]. The smaller number of gastric polyps can be treated with endoscopic snare resection, without surgery. If an early adenocarcinoma is found in a resected polyp and total resection is confirmed both endoscopically and microscopically, oncological endoscopic follow-up should include repeated EGDs at 1 year and 3 years following the initial polypectomy. If an incomplete early gastric cancer resection is unequivocally confirmed, or when effective endoscopic treatment is not feasible (i.e. total endoscopic polypectomy cannot be performed), gastrectomy and lymphadenectomy (involving regional lymph nodes) should always be considered, since the risk of lymph node involvement in these patients is very high.

## Conclusion

Gastric hyperplastic polyps are commonly considered to be insignificant in terms of potential malignant conversion; however, current studies report the presence of adenocarcinoma in some polyps. This is why symptomatic polyps, as well as polyps with focal dysplasia and early gastric cancer or larger than 10 mm, should be resected. Endoscopic treatment of gastric hyperplastic polyps with early cancer is considered to be sufficient if the endoscopic and microscopic completeness of the procedure is confirmed by the endoscopist and histopathologist. In such cases, surgical resection does not have to be performed and endoscopic surveillance is recommended. The follow-up should include the repeated EGD at 1 year and 3 years after polyp resection. Additionally, gastric mapping should be performed, in order to determine the phenotype of gastritis, with which gastric hyperplastic polyps are associated. Moreover, the eradication of *H. pylori* infection is recommended in order to eliminate the potential underlying cause of the main problem.
